# An Improved Systematic Approach to Predicting Transcription Factor Target Genes Using Support Vector Machine

**DOI:** 10.1371/journal.pone.0094519

**Published:** 2014-04-17

**Authors:** Song Cui, Eunseog Youn, Joohyun Lee, Stephan J. Maas

**Affiliations:** 1 School of Agribusiness and Agriscience, Middle Tennessee State University, Murfreesboro, Tennessee, United States of America; 2 Department of Computer Science, Texas Tech University, Lubbock, Texas, United States of America; 3 Department of Plant and Soil Science, Texas Tech University, Lubbock, Texas, United States of America; University of Ulm, Germany

## Abstract

Biological prediction of transcription factor binding sites and their corresponding transcription factor target genes (TFTGs) makes great contribution to understanding the gene regulatory networks. However, these approaches are based on laborious and time-consuming biological experiments. Numerous computational approaches have shown great potential to circumvent laborious biological methods. However, the majority of these algorithms provide limited performances and fail to consider the structural property of the datasets. We proposed a refined systematic computational approach for predicting TFTGs. Based on previous work done on identifying auxin response factor target genes from *Arabidopsis thaliana* co-expression data, we adopted a novel reverse-complementary distance-sensitive *n*-gram profile algorithm. This algorithm converts each upstream sub-sequence into a high-dimensional vector data point and transforms the prediction task into a classification problem using support vector machine-based classifier. Our approach showed significant improvement compared to other computational methods based on the area under curve value of the receiver operating characteristic curve using 10-fold cross validation. In addition, in the light of the highly skewed structure of the dataset, we also evaluated other metrics and their associated curves, such as precision-recall curves and cost curves, which provided highly satisfactory results.

## Introduction

Unraveling the gene regulatory networks is regarded as one of the fundamental problems challenging biologists [1]. Gene expression is systematically controlled by regulatory proteins known as transcription factors (TFs) that bind to specific cognate DNA sites known as transcription factor binding sites (TFBSs). Through interacting with other *cis*-elements, these TFs can function as repressors preventing transcription by inhibiting the activity of RNA polymerization complex either by directly binding to TFBSs or indirectly modifying transcription factor target genes (TFTGs). Transcription factors can also function as activators, which promote the expression of TFTGs. In addition to post-transcriptional gene regulations, there are also post-translational gene modification regulations, including biochemical alteration and RNA interference [2], [3]. However, the interplay among the corresponding TFs, TFBSs, and TFTGs remains the predominant mechanism governing the gene regulatory processes.

In order to circumvent the laborious biological experiments for screening TFBSs and their corresponding TFTGs, a number of computational algorithms have been proposed in the last decade on the basis of pre-established biological results [4]–[16]. Instead of directly searching for TFTGs, the majority of these algorithms focused on the nucleotide sequence information to screen potential TFBSs and ignored the structural property of DNA molecules. Local search-based algorithms, such as Gibbs sampling, have been applied on certain microorganisms with some success but lacked global optimality [4]–[7]. Position weight matrix-based approaches were popular but suffered greatly from high false-positive prediction rate and the independence assumption among different TFBSs [8]–[10]. Most recently, He *et al.* [11] refined the traditional *n*-gram profile algorithm based on the fact that a specific TF may bind on either a DNA strand or its reverse complement and produced satisfactory results. Following their work, Dai *et al.* [12] incorporated a positional signal into each potential TFBS and greatly improved prediction performances. Additionally, Meysman *et al.* [13] discussed a prediction algorithm using DNA structural information alone to predict TFBSs. *De novo* methodology-based predictions did not require any model training based on the prior knowledge of TFTGs thus showing its advantages in terms of computational cost and classification accuracy [14]–[16]. Unfortunately, all these approaches provided limited classification performances and failed to consider the dataset structure when interpreting the final results. Particularly, the method proposed by Dai *et al.* [12], which requires arbitrarily choosing thresholds for feature selection, could provide limited performances because the optimal threshold was not identified. Taking all these weaknesses into account, an improved new systematic computational approach for TFTG prediction was proposed in our study and produced great results.

## Materials and Methods

Using the well-documented information domain of the corresponding TFs, TFBSs, and TFTGs, we constructed a binary classifier based on support vector machines (SVM). A standard feature extraction, feature selection, model construction, and dataset testing paradigm was followed. The feature extraction region was limited to within 1000-bp upstream from the transcription start point. This frame was verified to contain the most amounts of TFBSs from previous biological studies [11], [12]. Once these 1000-bp sequences with identified class labels (TFTGs or non-TFTGs) were generated, they were then profiled by a new reverse-complementary distance-sensitive *n*-gram profiling (RCDSNGP) algorithm designed to better capture the patterns of potential conserved motifs and their corresponding positions relative to recognized TFBSs. For feature selection, we adopted Monte Carlo simulation based on information gain (IG) measurements to select features that have a *p*-value smaller than 0.01. Finally, each upstream sequence of either TFTGs or non-TFTGs was represented by a single data point in a multi-dimensional feature space and was later fed to SVM to build prediction models.

Feature extraction, selection, model training, and testing were performed on the basis of a 10-fold cross validation (10-fold CV). That is, the entire dataset is randomly and evenly split into 10 disjoint subsets. Each subset contains a proportion of TFTG sequences similar to that of the pre-split dataset, e.g. 19 TFTGs +260 non-TFTGs  = 279. The final result is calculated from a composite of 10 trials. Within each trial, a different subset of samples is selected for testing and the other nine subsets of samples are used for training. Feature extraction and selection are performed within the nine training subsets during each trial, thus, selected features are different in each trial.

### Datasets

The procedures for generating sequence datasets were described previously by Dai *et al.* [12]. In general, auxin response factors (ARFs) regulate their target genes by recognizing the primary conserved motif 'TGTCTC' or its reverse complement 'GAGACA' in the upstream region [17]. However, the presence of this conserved motif by itself may not guarantee that the corresponding sequence belongs to TFTGs. Goda *et al.* [18] used Affymetrix Genechip to investigate the gene expressions of *A. thaliana* treated with auxin and brassinosteroid and reported that only 186 out of 2787 genes containing the conserved motif ('TGTCTC' or 'GAGACA') in their 1000-bp upstream region were TFTGs.

By referring to the accession IDs verified by Goda *et al.* [18] and location information (transcription start point and chromosome ID) obtained from TAIR6 Arabidopsis Information Resource (ftp://ftp.arabidopsis.org/home/tair/Genes/TAIR6_genome_release), the 1000-bp gene upstream sequences of *A. thaliana* with the conserved motif (186 TFTGs +2601 non-TFTGs  = 2787) were extracted from the genome sequences (ftp://ftp.arabidopsis.org/home/tair/home/tair/Sequences/whole_chromosomes). The entire dataset can be downloaded from the Samuel Roberts Noble Foundation online supplementary data source (available via http://bioinfo.noble.org/manuscript-support/TF_Supp/dataset/).

### Feature extraction

Each upstream sequence has to be converted into a series of numerical values corresponding to its coordinates in a high-order feature space for SVM training and prediction purposes. An *n*-gram profiling algorithm was used previously to represent a sequence stream by a set of *n* continuous characters and their corresponding frequencies [19]. This approach is analogous to the *k*-mer approach used in other gene sequence studies [20].

Because of the double helix structure and base-pairing property of DNA, TFs may bind on either strand of a DNA molecule. Thus, a conserved motif and its reverse complement should be treated equally for each TF. In the light of this, He *et al.* [11] proposed the reverse-complementary n-gram profile (RCNP) algorithm, formalized as follows.

Definition 1 (RCNP): Given an *m*-length sequence *s* = *s*_1_, *s*_2_…*s_m_*, the RCNP of *s* is a set of *K* 2-tuples, denoted as RCNP(*s*) = {({*f*_1_, *r*_1_}, *c*_1_),({*f*_2_, *r*_2_}, *c*_2_)…({*f_K_*, *r_K_*}, *c_K_*)}, *f_k_* being a distinct *n*-gram, *r_k_* being the reverse complement of *f_k_*, and *c_k_* being the sum of frequency counts of *f_k_* and *r_k_* in *s*. Additionally, {*f_k_*, *r_k_*} (*k* = 1, 2…*K*) includes all possible combinations of an *n*-gram and its reverse complement in *s*.

The essence of RCNP was that an occurrence of either an *n*-gram or its reverse complement will be counted equally as one increment of that feature ({*f_i_*, *r_i_*}). In addition, by limiting the feature extraction region within a finite window evenly neighboring the center motif (such as 100-bp window size with 50 bp on each flank), He *et al.* [11] considered the presence of other possible synergic TFBSs within the window beside the center motif. This approach was based on the assumption that the closer an *n*-gram is to the primary TFBSs, the stronger its influence on regulating TF binding processes. An optimal area under curve (AUC) value of 0.8949 was obtained on a similar dataset using this RCNP algorithm [11].

Immediately after He *et al.*'s work, Dai *et al.* [12] expanded the RCNP algorithm into a reverse-complementary position-sensitive *n*-gram profile (RCPSNP) algorithm by incorporating a positional information parameter and a position-sensitive parameter into the RCNP. The position sensitive parameter was introduced to mainly account for the possibility that two identical *n*-grams extracted from a certain window flanking the center motif on the same DNA strand may have similar impacts on regulating TF binding processes regardless of their positional differences. This feature generation scheme yielded an AUC value of 0.73 for the receiver operating characteristic (ROC) curve [12].

In this study, we propose an improved feature generation algorithm. Studies have shown the existence of a composite structure containing constitutive elements adjacent to the 'TGTCTC' binding site for ARFs [17], [21]. As a result, the auxin inducibility was likely affected incrementally by multiple elements. In addition, their contribution differences should be related with the distance from each element to the primary TFBS. None of the previous studies investigated the impact differences between the upstream-region elements and the downstream-region elements around the primary binding sites. Therefore, it was not logical to incorporate each *n*-gram with a signed integer representing its direction and distance relative to the primary TFBS as proposed by Dai *et al.* [12]. Considering all factors described above, we introduced the reverse-complementary distance-sensitive *n*-gram profile (RCDSNGP), formalized as follows.

Definition 2 (RCDSNGP): Given an *m*-length sequence *s* = *s*_1_, *s*_2_… *s_i_*…*s_i+j_*…*s*_m_, the RCDSNGP of *s* with respect to a *j*-length reference subsequence *x* = *s_i_*…*s_i+j−1_* is a set of *K* 2-tuples, denoted as RCDSNGP(*s*) = {({*f*_1_, *r*_1_, *d*_1_}, *c*_1_),({*f*_2_, *r*_2_, *d*_2_}, *c*_2_)…({*f_K_*, *r_K_*, *d_K_*}, *c_K_*)}, *f_k_* being a distinct *n*-gram, *r_k_* being the reverse complement of *f_k_*, *d_k_* being the relative distance parameter, and *c_k_* being the sum of frequency counts of *f_k_* and *r_k_* with the same *d_k_* relative to *x* in *s*. Additionally, {*f_k_*, *r_k_*, *d_k_*} (*k* = 1, 2…*K*) include all possible combinations of an *n*-gram, its reverse complement, and its distance to *x* in *s*.

If we denote either *f_k_* or *r_k_* as an *n*-gram, *g* = *s_t_, s_t+1_*…*s_t+n−1_* (*n*−1<*t*+*n*−1<*i* or *m*−*n*+2>*t*>*i*+*j*−1), then its relative distance to *x* is calculated as follows.
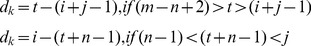
(1)


Each set ({*f_k_*, *r_k_*, *d_k_*}) within a 2-tuple of an RCDSNGP is a reverse-complementary distance-sensitive *n*-gram (RCDSNG), synonymously a feature in our study.

By adopting RCDSNGP, an occurrence of either an *n*-gram or its reverse complement with the same distance to the central TFBS will be counted equally as one increment of that feature ({*f_k_*, *r_k_*, *d_k_*}).

Table 1 demonstrates an example of an RCDSNGP of a given sequence with respect to a designated reference subsequence.

**Table 1:** An example of a reverse-complementary distance-sensitive *n*-gram profile (RCDSNGP) representation with *n* = 4, 5, and 6 for a given sequence (AAGCTT**GAGACA**CAGCT) with the reference subsequence marked in bold*.

Length of *n*-gram (*n*)	Reverse-complementary distance-sensitive *n*-gram (RCDSNG), or feature	Frequency count
*n* = 4	{AAGC, GCTT, 1}	1
	{AGCT, AGCT, 2}	2
	{AAGC, GCTT, 3}	1
	{CAGC, GCTG, 1}	1
*n* = 5	{AAGCT, AGCTT, 1}	1
	{AAGCT, AGCTT, 2}	1
	{AGCTG, CAGCT, 1}	1
*n* = 6	{AAGCTT, AAGCTT, 1}	1

^*^Given an *m*-length sequence *s* = *s*_1_, *s*_2_… *s_i_*…*s_i+j_*…*s*_m_, the RCDSNGP of *s* with respect to an *j*-length reference subsequence *x* = *s_i_*…*s_i+j−1_* is a set of *K* 2-tuples, denoted as RCDSNGP(*s*) RCDSNGP(*s*) = {({*f*_1_, *r*_1_, *d*_1_}, *c*_1_),({*f*_2_, *r*_2_, *d*_2_}, *c*_2_)…({*f_K_*, *r_K_*, *d_K_*}, *c_K_*)}, *f_k_* being a distinct *n*-gram, *r_k_* being the reverse complement of *f_k_*, *d_k_* being the relative distance parameter, and *c_k_* being the sum of frequency counts of *f_k_* and *r_k_* with the same *d_k_* relative to *x* in *s*. Each set in a 2-tuple ({*f_k_*, *r_k_*, *d_k_*}) is a reverse-complementary distance-sensitive *n*-gram (RCDSNG), or a feature in our study. This RCDSNGP representation was adopted for all training sequences. For testing processes, each sequence was converted to RCDSNGP first, and then represented according to the selected RCDSNGs generated from the training datasets, including those with zero count.

### Feature selection

We used *n*-grams of *n* = 4–9 for profiling each upstream sequence using RCDSNGP algorithm because *n* = 4–9 were verified (data not shown) to give optimal performances and can be handled efficiently in a moderate computing environment. Given the maximum distance *d_max_*, a total number of *d_max_*×4*^n^* features at most can be generated for each *n*, which is less than half of the number produced by Dai *et al*. [12].

Given a *d_max_*, 10 trials were conducted to generate the final result (10-fold CV). Within each trial, a separate IG-based feature ranking was used on the nine training subsets [22]. The IG measure is based on information theory [23], which calculates the entropy differences before and after observing a specific feature. The entropy of the set *S*, which contained e.g. *N* = 2509 (168 TFTGs +2341 non-TFTGs  = 2509) upstream sequences and two distinct class labels 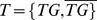 for the TFTGs and non-TFTGs (number of classes *y* = 2), can be calculated as
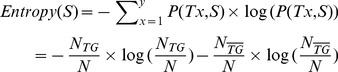
(2)where 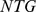 and 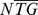 are the numbers of sequences in *S* belonging to TFTGs and non-TFTGs, respectively.

After observing a specific feature *f*, we can partition the original set *S* into two distinct subsets: 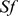, a set of upstream sequences containing *f*; 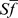, a set of upstream sequences without *f*. Thus, *S* = {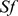, 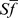} and the number of classes is *y* = 2. The entropy of *S* with respect to *f* is evaluated as
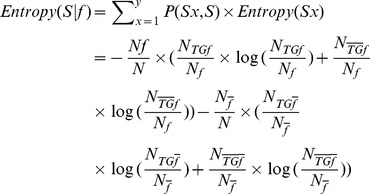
(3)where numbers of upstream sequences with at least one occurrence of feature *f* and no occurrence of *f* are denoted by 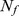 and 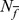, respectively; numbers of upstream sequences belonging to TFTGs with at least one occurrence of feature *f* and no occurrence of *f* are denoted by 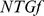 and 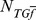, respectively; and, 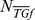 and 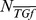 represent the corresponding numbers of non-TFTGs. Finally, the IG obtained by dividing *S* according to *f* is calculated using:
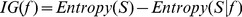
(4)and a higher IG value implies greater importance of a given feature for representing a specific sequence.

Instead of ranking features based on IGs and subjectively choosing the cutoff value, we further evaluated each feature using Monte Carlo simulations [24], [25]. This approach provides information on whether we can statistically differentiate samples from two classes on a basis of a given feature. For each feature, we shuffled class labels (TFTGs or non-TFTGs) 10 000 times without changing either the feature count in each sequence or the total number of sequences in each class. A new IG was calculated for each shuffling, thus, 10 000 IGs were obtained for each feature. The *p*-value for a specific feature was calculated according to
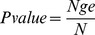
(5)where 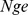 represents the number of shuffling that gave new IG values greater than or equal to the original one and *N* is the total number of shuffling (*N* = 10 000). The smaller the *p*-value is, the stronger the contribution the feature would provide to differentiate a sample between two classes. In our study, features were considered important at *p*<0.01 level.

### Data representation

Within each trial, we selected *F* features whose *p*-values were smaller than 0.01 from the total of 493 781 all possible features (number of RCDSNGs obtained from all sequences). Each 1000-bp upstream sequence was represented by an RCDSNGP consisting of these *F* features and their corresponding occurrences. The whole set of *N* sequences (e.g. *N* = 168 TFTGs +2341 non-TFTGs  = 2509) were then represented by an *N*×(*F*+1) matrix (1 extra column for class labels).

### Training and testing

Support vector machine-based approaches have been widely used in various problem domains, such as bioinformatics [26]–[28]. They often outperformed many other classification algorithms [29]. We adopted SVM in our study using the LIBSVM [30], which is based on sequential minimal optimization. The general concept of SVM is given below.

For model training, given a set of vector-label pairs 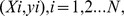 where 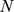 is equal to the number of upstream sequences (e.g. *N* = 2509); 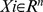, where *n* is the dimension of 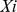, equal to the number of selected features; 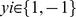 where 1 corresponds to TFTGs and -1 corresponds to non-TFTGs, the support vector machine computes the solution to the optimization problem formalized below:
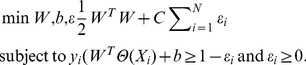
(6)


This is equivalent to finding the maximum-margin hyperplane that separates TFTG samples (labeled as 1) and non-TFTG samples (labeled as -1) with minimized measures of errors. Predictions are made based on the geometric location of an unknown sample when fed into the model. A label is assigned to a sample according to which side of the hyperplane it resides. In order to obtain better classification accuracies, data are often projected into a high-dimension feature space with a kernel function. We evaluated other possible kernels in addition to the linear kernel suggested by Dai *et al.* [12]. Particularly, we performed a grid (factorial) search [28] for an optimal combination of a penalty factor *C* of SVM and a kernel width 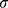 for the Gaussian radial basis function (RBF) kernel.

### Performance measure

First, we measured the traditional accuracy defined as
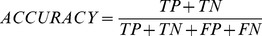
(7)where all parameters are defined in Table 2. Accuracy provides a direct and simple way of evaluating performances, however, it is highly sensitive to data distribution [29]. In our study, if a classifier predicts every TFTG sequence as a non-TFTG sequence, we can still obtain an accuracy of 0.9333 due to the fact that more than 93 percent of the sequences belong to non-TFTGs. Thus, other evaluation metrics are warranted, including precision, recall, and *F*_1_ [29] defined as follows.

**Table 2:** Confusion matrix for performance evaluation with positive class label (+1) denoting transcription factor target gene (TFTG) and negative class label (-1) denoting non-TFTG.

		True class label	
		+1	−1
Predicted class label	+1	TP++	FP^−+^
	−1	FN^−−^	TN^+−^

++ , ^−+^, ^−−^, ^+−^ denote true positive, false positive, false negative, and true negative, respectively.



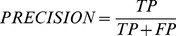
(8)

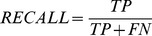
(9)




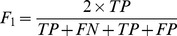
(10)


Furthermore, we adopted the ROC curve technique, which demonstrated satisfactory performances across different classifiers and datasets from previous studies [12]. Some recent studies argued that the ROC approach tends to provide an over-optimistic evaluation on highly imbalanced datasets [30]. Thus, we also examined precision-recall (PR) curves, which appeared more informative and valid than ROC curves on skewed datasets [31]. The AUC value was calculated on both the ROC curve (ROC-AUC) and the PR curve (PR-AUC) to summarize the classification results. To better visualize the misclassification costs and statistical significances, cost curves [32] were also evaluated.

Dai *et al.* [12] reported that a window size of 200 bp (100 bp on each flank around the central TFBS, ‘TGTCTC’ or ‘GAGACA’) provided the best ROC-AUC value. In our study, a new feature generation scheme was adopted. Therefore, we evaluated different maximum distances (*d_max_* = half of the window size) on each flank of the central TFBSs, including *d_max_* = 25, 50, 75, 100, 125, 150, 175, and 200 bps.

To summarize, we evaluated eight different *d_max_* settings within each trial on the basis of 10-fold CV. Within each *d_max_* setting during each trial, an IG-based *p*-value selection procedure was adopted to select the important (*p*<0.01) features based on the training dataset (nine folds out of 10). Immediately following that, we located the optimal combination of *C* for SVM and σ for the RBF kernel using the grid (factorial) search. Thus, the optimal combination of features, *C*, and σ depended on the training dataset within each trial. The final results, including accuracy, precision, recall, and *F*_1_ as well as the corresponding AUC values were generated from the combined classification results containing all 2787 sequences according to different *d_max_* settings.

## Results

### Extraction of features from sequences

Using a maximum distance *d_max_* = 150 and *n* = 4–9 for building RCDSNGP, 2 455 252 unique features were generated from 1000-bp upstream of 2787 sequences (186 TFTGs +2601 non-TFTGs). We eliminated singleton features that contain only 1 occurrence across all sequences to reduce the feature size down to 735 624. The same technique was applied to all other settings of *d_max_* as well.

### Selection of representative features

Information gain calculated for each feature was used in Monte Carlo simulation to generate a *p*-value for that feature. We selected those features that had *p*-values smaller than 0.01. For different maximum distances, *d_max_* = 25, 50, 75, 100, 125, 150, 175, and 200, we selected 893, 1870, 2732, 3502, 4255, 4949, 5622, and 6136 unique features, respectively on the basis of 10-fold CV (Table 3). Table 4 lists the top 20 features ranked by their *p*-values with corresponding IGs, when *d_max_* = 150.

**Table 3:** Number of unique features (union of selected features with *p*-value <0.01 based on 10-fold cross validation) and classification performances [evaluated as accuracy, precision, recall, *F*_1_, area under the curve (AUC) value of receiver operating characteristic (ROC) curve, and AUC value of precision-recall (PR) curve] affected by the maximum distance on each flank from the central binding site (*d_max_*) based on 10-fold cross-validation on transcription factor target gene prediction using reverse-complementary distance-sensitive *n*-gram profile algorithm with *n* = 4–9 and support vector machine with Gaussian radial basis function kernel.

d_max_	Unique Feature	Accuracy	Precision	Recall	F_1_	ROC-AUC	PR-AUC
25	893	0.9476	0.6923	0.3871	0.4966	0.7202	0.4180
50	1870	0.9541	0.7544	0.4624	0.5733	0.7562	0.5286
75	2732	0.9559	0.7739	0.4785	0.5914	0.7739	0.5554
100	3502	0.9566	0.7519	0.5215	0.6159	0.7720	0.5808
125	4255	0.9580	0.7899	0.5054	0.6164	0.7640	0.5646
150	4949	0.9602	0.8319	0.5054	0.6288	0.7626	0.5690
175	5622	0.9587	0.8034	0.5054	0.6205	0.7673	0.5639
200	6136	0.9580	0.7805	0.5161	0.6214	0.7664	0.5879

**Table 4:** The top 20 smallest *p*-value reverse-complementary distance-sensitive *n*-grams (RCDSNGs; *n* = 4–9) selected as representative features with their information gain (IG) values and *p*-values in the 1000-bp upstream of 186 transcription factor target genes (TFTGs) and 2601 non-TFTGs, when the maximum distance (half of the window size) on each flank of the central transcription factor binding site *d_max_* = 150.

Ranking	RCDSNG (feature)	IG value	*p*-value
1	{ACACGT, ACGTGT, 4}	0.001885	0
2	{CGAGAA, TTCTCG, 82}	0.001884	0
3	{AATATAA, TTATATT, 52}	0.001884	0
4	{ACTTCC, GGAAGT, 30}	0.001880	0
5	{ACACC, GGTGT, 44}	0.001880	0
6	{GTAC, GTAC, 39}	0.001701	0
7	{CAAACA, TGTTTG, 149}	0.001707	0
8	{AAAAATA, TATTTTT, 44}	0.001707	0
9	{AGTAT,ATACT, 51}	0.001713	0
10	{ATGATTA, TAATCAT, 130}	0.001656	0
11	{ACTTC, GAAGT, 30}	0.001516	0
12	{CTAAC, GTTAG, 91}	0.001476	0
13	{ACAAATA, TATTTGT, 71}	0.001463	0
14	{ATACG, CGTAT, 49}	0.001463	0
15	{AAAACC, GGTTTT, 75}	0.001463	0
16	{AAAGACA, TGTCTTT, 117}	0.001463	0
17	{TAAAACA, TGTTTTA, 85}	0.001463	0
18	{AGTATA, TATACT, 124}	0.001458	0
19	{AATGTG, CACATT, 43}	0.001412	0
20	{ATACCC, GGGTAT, 16}	0.001412	0

### Classifier performances

We implemented our own 10-fold CV SVM with Python programming language on the basis of LIBSVM. Using the *p*<0.01 threshold, models were constructed based on different combinations of maximum distances and kernels (polynomial, RBF, sigmoid, and linear kernels), with *n*-grams of *n* = 4–9. The RBF kernel provided the best performances regardless of measurement metrics used. In our study, the best accuracy (0.9602), precision (0.8319), and *F*1 (0.6288) values were obtained with *d_max_* = 150. The best recall (0.5215), ROC-AUC (0.7739), and PR-AUC (0.5879) values were obtained with *d_max_* = 100, 75, and 200, respectively (Table 3).

**Figure 1:**
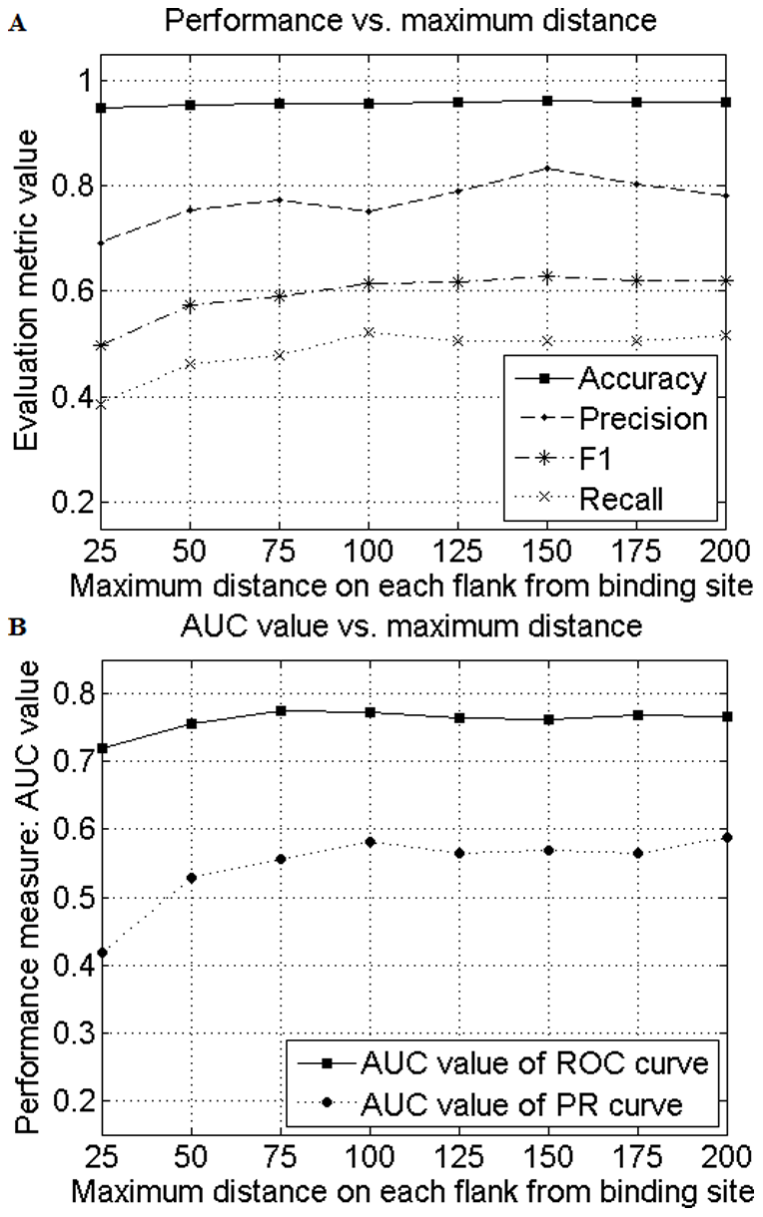
Performances of transcription factor target gene prediction affected by the maximum distance on each flank from the binding site (*d_max_*) based on 10-fold cross-validation using reverse-complementary distance-sensitive *n*-gram profile algorithm with *n* = 4–9 and support vector machine with Gaussian radial basis function kernel. (A) Performance evaluation metric (accuracy, precision, recall, and *F*_1_) values versus *d_max_* on each flank from the central binding site. (B) Area under the curve (AUC) value of receiver operating characteristic (ROC) curve and precision-recall (PR) curve versus *d_max_* on each flank from the central binding site.

Figure 1a shows the response of accuracy, precision, recall, and *F*_1_ values versus *d_max_*. Accuracy fails to provide adequate information on evaluating the minority samples (TFTGs). Combining different evaluation metrics tends to provide comprehensive assessment of classification on imbalanced datasets. When *d_max_* = 25, our model suffered from low recall rate because of its poor accuracy when classifying the positive samples (TFTGs). Starting from *d_max_* = 50, a boost in performances was observed in recall and *F*1 values as a result of increased accuracy in predicting positive samples. At *d_max_* = 150, the model reaches the highest accuracy value of 0.9602. The best precision value is 0.8319 when *d_max_* = 150 and declines a little as *d_max_* increases. Starting from *d_max_* = 100, recall values are above 0.5 and peak at *d_max_* = 100 with a value of 0.5215. Likewise, the *F*_1_ scores are above 0.6 when *d_max_* is bigger or equal to 100 and reach the greatest value of 0.6288 when *d_max_* = 150. Figure 1b shows an AUC-versus-*d_max_* curve based on both the ROC curve and the PR curve. The ROC-AUC value arrives at 0.7739 when *d_max_* = 75. A slight decrease is detected when *d_max_*>100. Furthermore, by varying *d_max_* the PR-AUC value responds more obviously than the ROC curve. Beginning at *d_max_* = 100, our model produces above-0.56 PR-AUC values, which gradually decrease from *d_max_* = 100 to *d_max_* = 125 and peaks at 0.5879 when *d_max_* = 200. Overall, a 150-*d_max_* setting is likely to give a superior performance with limited complexity of computation compared to other maximum distance settings. Although it fails to produce the optimal AUC values for either ROC or PR curve, it provides the best accuracy, precision, and *F*_1_ values. Considering the fact that most *d_max_* value settings perform well in detecting the correct negative samples (non-TFTGs), the model that is most capable of identifying the correct positive samples (TFTGs; high precision value) yields the best results. A maximum distance *d_max_* = 200 provides great PR-AUC values. However, it adds great computational cost for feature generation and selection processes (possibly 50× 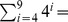 17 472 000 more features) and some of its performance metrics are even worse than *d_max_* = 150. Therefore, we conclude that features within a maximum distance *d_max_* = 150 around central TFBSs contain sufficient information for making accurate prediction on TFTGs.

**Figure 2:**
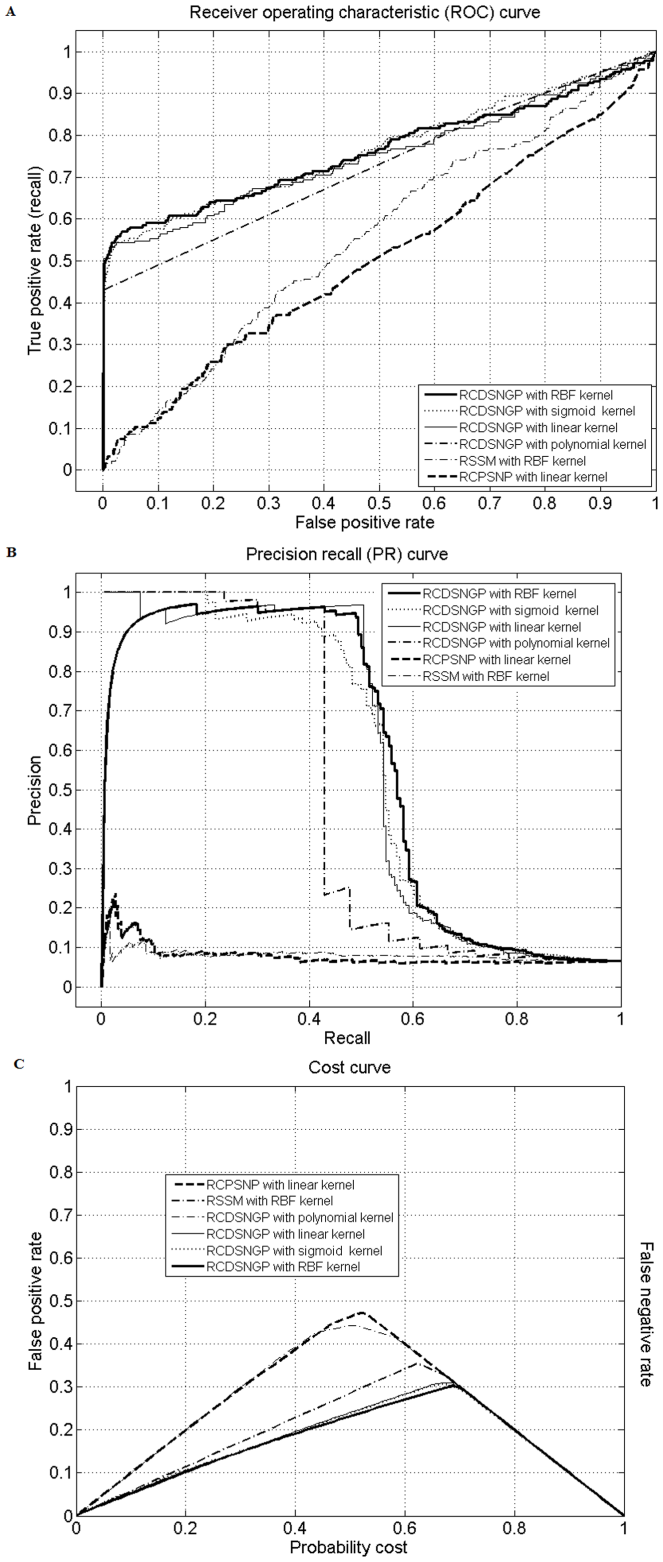
Classification performances using the optimal maximum distance on each flank from the binding site (*d_max_* = 150). (A) Receiver operating characteristic (ROC) curve, (B) precision-recall (PR) curve, and (C) cost curve of the 10-fold cross-validation on transcription factor target gene prediction using reverse-complementary distance-sensitive *n*-gram profile (RCDSNGP) algorithm with *d_max_* = 150 and *n* = 4–9 based on different support vector machine (SVM) kernels, reverse-complementary position-sensitive *n*-gram profile (RCPSNP) algorithm using linear-kernel SVM, and Position-Specific Scoring Matrices (PSSM)-based approach.

In order to show the advantages of our proposed RCDSNGP algorithm compared to other algorithms [12], we also examined the ROC, PR, and cost curve as well as precision, recall, and *F*_1_ value generated by different algorithms [12] based on the same 10-fold CV split. Additionally, comparisons were made among classifiers with different kernels. Figure 2a indicates that RCDSNGP-based model with RBF kernel outperformed all other models because it generates a curve that is closer to the perfect classification point (0,1) in the ROC curve compared with all others. Interestingly, the polynomial kernel produced bad result because it predicted each sample with a fixed score of -1. Classifiers that dominate in ROC space should dominate in PR space as well [33]. This is vividly presented by Figure 2b. Furthermore, our study highlighted the drawbacks of over-dependency on the ROC-AUC value when evaluating the classification performances. Although the difference in ROC-AUC values was relatively small (0.7626 versus 0.5055) between RCDSNGP and RCPSNP algorithms, the difference in PR-AUC values was remarkable (0.569 versus 0.0773).

Finally, we evaluated the cost curve of each model. The cost curve, which emphasizes the expected misclassification cost for performance measure, is proposed to better visualize the misclassification cost and compare performances on the basis of statistical significance [32]. Each data point in the ROC curve gets mapped to a distinct straight line (cost line) in the cost curve by connecting point (0, FP) and point (1, FN). Multiple points in the ROC space would generate several cost lines and form a lower envelope, which is shown in Figure 2c. The *x*-axis, denoted as probability cost, includes all possible percentage values of positive samples (0 to 1). It represents the proportion of positive samples when the classifier is deployed. Thus, at *x* = 0 (no positive samples), the only possible misclassification errors are FPs. Likewise, at *x* = 1 (no negative samples), the only possible misclassification errors are FNs. The straight line connecting these two points represents the trend of misclassification cost as percentage of positive samples varies. The lower envelope generated by a non-discrete classifier such as SVM or Artificial Neural Network is the counterpart for the upper convex hull of the ROC curve. At each probability cost value, the closer the curve is to the *x*-axis, the better the classifier performs (a lower expected cost). As presented in Figure 2c, the RCDSNGP-based model with RBF kernel has the lowest cost from 0 to 68 percent of positive samples. Additionally, it is also verified to be different (*p*<0.05) from RCPSNP-based model within the 4.8 to 65 percent range of positive samples using the method proposed by Drummond and Holte [32]. In our study, the dataset contains 6.7 percent of positive samples, which is within the 4.8 to 59 percent range, thus our model outperformed RCPSNP-based model in terms of expected cost.

To summarize, using new feature generation and selection strategies to predict TFTGs of ARFs in *A. thaliana* based on published datasets [12], we drastically increased classification performances. Our best result was obtained when *d_max_* = 150 using the RBF kernel based on an average of 2395 features. We adopted the ROC measure for efficacy evaluation and obtained an ROC-AUC value of 0.7626 (SE = 0.0021), accuracy value of 0.9602 (SE = 0.0023), precision value of 0.8319 (SE = 0.0331), recall value of 0.5054 (SE = 0.0231), and *F*_1_ score of 0.6288 (SE = 0.0211; Table 3), which were higher than the best result reported by Dai *et al.* (12; ROC-AUC = 0.73, accuracy  = 0.69, precision  = 0.3684, recall  = 0.1129, and *F*_1_ = 0.1728) based on the same dataset but with a different 10-fold CV split.

Additionally, Dai *et al.* [12] reported the performance of a Position-Specific Scoring Matrices (PSSM)-based approach using the cluster-buster algorithm [34], which only yielded an ROC-AUC value of 0.51. We implemented a traditional approach based on the position frequency matrix method using a similar feature encoding algorithm explained in Youn *et al.* [26]. Each sequence was parsed according to a *d_max_* = 150 setting and the sequence conservation was evaluated using a four (four nucleotides) by 300 (2×*d_max_*) position frequency matrix (150-bp flanking each side of the primary conserved motif). At each residue (1 out of 300), we considered 20-bp window size (left:10, right: 10) to construct the frequency count for each nucleotide. The standard SVM-based training and testing was performed based on generated PSSMs. Likewise, the algorithm only provided an ROC-AUC value of 0.5569 and a PR-AUC value of 0.0801 using the same 10-fold CV split as our RCDSNGP algorithm (Table 5). The performance curves based on PSSM are also presented in Fig. 2.

**Table 5:** Classification performances [evaluated as accuracy, precision, recall, *F*_1_, area under the curve (AUC) value of receiver operating characteristic (ROC) curve, and AUC value of precision-recall (PR) curve] using different feature encoding algorithms with optimal parameter settings for SVM and *d_max_* = 150, including reverse-complementary distance-sensitive *n*-gram profile (RCDSNGP), reverse-complementary position-sensitive *n*-gram profile (RCPSNP), and a Position-Specific Scoring Matrices (PSSM)-based algorithms.

Feature Encoding Algorithm	Accuracy	Precision	Recall	*F*_1_	ROC-AUC	PR-AUC
RCDSNGP	0.9602	0.8319	0.5054	0.6288	0.7626	0.5690
RCPSNP	0.9300	0.2000	0.0161	0.0299	0.5055	0.0773
PSSM	0.9332	NAN	0	0	0.5569	0.0801

Furthermore, we also implemented the RCPSNP algorithm proposed by Dai et al [12] using the optimal settings (e.g. *n* = 4–9, linear-kernel SVM) and applied it to the same 10-fold CV split as our RCDSNGP algorithm, which yielded an ROC-AUC value of 0.5055, PR-AUC value of 0.0773, accuracy value of 0.9300, precision value of 0.2000, recall value of 0.0161, and *F*_1_ score of 0.0299 (Table 5). Our classifier generated points much closer to the perfect classification point (0,1) in the ROC curve than those generated by RCPSNP algorithm (Fig. 2a; 12). Most importantly, considering that traditional metrics for measuring classification performances tended to provide deceiving or inadequate information of imbalanced datasets, we also evaluated other metrics and their corresponding curves such as PR and cost curves, which showed greatly improved results as well.

The detailed model files, 10-fold CV datasets represented as matrices, and classification results are available at the supplementary online data source.

## Discussion

Understanding the mechanism of gene regulatory network is a challenging task. As of today, there is still much uncertainty in identifying the corresponding TFBSs and TFTGs. More TF and TF-dependent target gene regulation studies are required to evaluate the biological function and mechanism of more gene regulation players. The activity and affinity of TF would be the ultimate balanced result of the various check points of biological regulation. The binding efficiency of TF to its corresponding TFBS is regulated by various factors, including TF synthesis, ligand binding to the TFs, and DNA binding mechanism through post-translational modifications such as phosphorylation and glycosylation of the TFs. In addition, the DNA binding process, dimerization, and interactions with cofactors for the functional complex formation are important parameters controlling the TF activity [35], [36]. As more information of the interplay among TF, its corresponding TFBS, and TFTG accumulates, it could be possible to understand the precise TFTG expression affected by different TFs. A number of computational approaches that rely on well-known TFBSs have been proposed, but a majority of these algorithms suffered from high FP rate [12], [37]. Therefore, much effort was put on reducing FP rates and increasing prediction accuracies [12], whereas the importance of the dataset structure was ignored. In our study, only 186 out of 2787 genes that all contain the binding site ('TGTCTC' or 'GAGACA') in their 1000-bp upstream region were TFTGs. If our model correctly predicted all negative samples (non-TFTGs) and miss-predicted all positive samples (TFTGs), it still yielded an accuracy value of 0.9333 (2601/2787) but with precision value undefined and zero values for both recall and *F*_1_ score. Therefore, minimizing the FP rate or maximizing the accuracy contributes little to improving overall performances when analyzing a highly skewed dataset. It is important to analyze different evaluation metrics to better assess the classification performances.

We deployed a novel feature extraction method (RCDSNGP) that incorporated a relative distance parameter into each feature to count for the positional information of each motif relative to the central TFBS. For feature selection, we adopted a Monte Carlo simulation-based statistical approach rather than arbitrarily choosing thresholds. We compared our results with the RCPSNP-based approach [12] on the same dataset. Our best model achieved an accuracy of 0.9602 and an ROC-AUC value of 0.7627 when *d_max_* = 150 compared with 0.69 and 0.73 reported by Dai *et al.* [12], respectively. Dai *et al.* introduced three parameters for constructing RCPSNP, including a number of *n*-grams *C* (analogous to our maximum distance *d_max_*), a top *F* representative features based on IG, and a position sensitive factor *P* (the identical *n*-grams located within a *P*-bp region neighboring the central binding site are counted equally). The best result was obtained when *n* = 4–9, *C* = 100, *P* = 100, and *F* = 1000. Their detailed results containing prediction scores can be found in their supplementary web data [12]. Moreover, little positional information is considered when *C* equals *P*. In other words, RCPSNP behaves almost the same as RCNP [11] when *C* and *P* hold the same value. The significant performance increase based on our RCDSNGP algorithm indicated that ARFs function by recognizing multiple consensus motifs that might be co-occurring TFBSs or subsequences functioning coordinately. More importantly, the relative distance from each motif to the binding site should always play an important role in the gene regulation process. The structural complexities of protein and DNA may result in a type of mutual recognition that relies more on the distance from the conserved motif to the TFBS, regardless of where the motif lies (downstream or upstream of the central TFBS). The PSSM-based approaches may be useful for TFTG identification when more associated TFBSs are known.

Identifying patterns of other potential TFBSs and the binding property of ARFs by enumerating all possible *n*-grams is a computationally expensive work. The complexity becomes even greater when a distance parameter is included. Therefore, better feature selection methods become necessary. We employed a statistical systematic approach. Based on a given feature, two class samples are different from each other if, and only if, the probability of obtaining a bigger IG value than original is below a certain level (*p*-value). This probability value is obtained using Monte Carlo simulations [38]. We verified that our feature selection algorithm is robust for a range of *p*-values (between 0.005 and 0.01). However, when the *p*-value becomes bigger, feature number increases drastically, which greatly increases computational cost. Moreover, we also evaluated a number of important features that have a *p*-value smaller than the 0.01 threshold versus different *d_max_* values (data not shown). The slope of the curve reached the maximum value between *d_max_* = 25 and *d_max_* = 50 and began to dwindle when *d_max_*>50, suggesting that flank regions closer to the core motif contain more important features for predicting TFTGs.

Precision-recall curve is used in information retrieval as an alternative to ROC curve when analyzing imbalanced datasets [33]. Optimal prediction models tend to generate curves close to the upper-left corner in the ROC curve and upper-right corner in the PR curve. Likewise, the cost curve is introduced to measure the performances by varying class probabilities to generate confidence intervals [32]. Regardless of which curve was used, RCDSNGP-based approaches using RBF kernel demonstrated significant advantages over the RCPSNP-based approach [12]. The polynomial kernel somehow yielded much poorer performances than others. Cost curves verified the similar effects by showing that superior models always generate a lower envelope curve than inferior ones. In other words, superior models always have significantly lower misclassification cost within a certain percentage range of positive samples.

Taken altogether, the RCDSNGP algorithm combined with statistical feature selection methods provides an efficient and highly accurate way to predict TFTGs on the basis of well-studied TFBSs. We believe that this improved methodology can be employed when analyzing other species besides *A. thaliana*. It might also provide new insights into the understanding of gene regulatory networks.
